# Life in High Salt Concentrations with Changing Environmental Conditions: Insights from Genomic and Phenotypic Analysis of *Salinivibrio* sp.

**DOI:** 10.3390/microorganisms7110577

**Published:** 2019-11-19

**Authors:** Jojy John, Vinu Siva, Kumari Richa, Aditya Arya, Amit Kumar

**Affiliations:** 1 International Research Centre for Climate Change Studies, Sathyabama Institute of Science and Technology, Old Mahabalipuram Road, Chennai 600119, India; 2 Amazing Biotech Private Limited, Marakkanam, Tamil Nadu 604303, India; 3 School of the Environment, Florida A&M University, Tallahassee, FL 32307-3103, USA; 4 Pathfinder Research and Training Foundation, 30/7, Knowledge Park III, Greater Noida, Gautam Budh Nagar, Uttar Pradesh 201308, India

**Keywords:** halophile, UV-tolerance, heavy metals, metabolic pathways, genome

## Abstract

Life in salt pans with varying chemical compositions require special adaptation strategies at both the physiological and molecular level. The Marakkanam salt pan in South India is characterized with a high fluctuation in salinity (19–490 ppt), Ultravioletradiation, and heavy metal concentrations. Several bacterial species have been isolated and identified in the view of phylogenetic analysis and for the subsequent production of industrially important enzymes. However, limited information exists on the genomic basis of their survival under variable environmental conditions. To this extent, we sequenced the whole genome of the *Salinivibrio* sp. HTSP, a moderately halophilic bacterium. We analysed the physiological and genomic attributes of *Salinivibrio* sp. HTSP to elucidate the strategies of adaptation under various abiotic stresses. The genome size is estimated to be 3.39 Mbp with a mean G + C content of 50.6%, including 3150 coding sequences. The genome possessed osmotic stress-related coding sequences, and genes involved in different pathways of DNA repair mechanisms and genes related to the resistance to toxic metals were identified. The periplasmic stress response genes and genes of different oxidative stress mechanisms were also identified. The tolerance capacity of the bacterial isolates to heavy metals, UV-radiation, and salinity was also confirmed through appropriate laboratory experiments under controlled conditions.

## 1. Introduction

Solar salterns represent an extreme environment with five to ten times saltier water than seawater, in addition to high ultraviolet (UV) radiation and low oxygen concentration [1]. These extreme conditions make it hard for the living organisms to thrive [2,3]. Salt pans in South India were observed to have a high content of heavy metals such as nickel (Ni), lead (Pb), copper (Cu), Zinc (Zn), Cadmium (Cd), Cobalt (Co), Manganese (Mn), Magnesium (Mg), and fluorides (F) [4,5]. Previous studies at these salt pans have revealed the presence of halotolerant and halophilic microbes [4,6]. The elucidation of bacterial genomics from these salt pans could provide sufficient understanding of their evolution under harsh and variable environmental conditions. Hence, we have isolated the *Salinivibrio* sp. from the Marakkanam Saltpan, and analysed its genomic and phenotypic attributes. 

Salinivibrios are moderately halophilic bacteria inhabiting a wide range of ecological habitats including hypersaline lakes, marine salterns, salt spring, saltpan, soda lakes, stromatolites, saline soil, and salt mines, salted meats, and brines, etc. [7,8,9,10]. They have adapted to thrive under halophilic conditions, and have been used as model organisms for unraveling halo-tolerance mechanisms [11]. The novel genus, *Salinivibrio,* in the family, *Vibrionaceae,* within the class, Gammaproteobacteria, was proposed by Mellado et al. [12] to accommodate the *Vibrio costicolus* (later renamed as *V. costicola* [13]) described by Smith (1938) based on *16S rRNA* gene phylogeny [12]. Upon revision of taxonomic position, *V. costicola* was renamed as *Salinivibrio costicola.* Soon after, three new subspecies of *S. coticola* (*S. costicola* subsp. *vallismortis* (now identified as *S. proteolyticus*), *S. costicola* subsp. *alkaliphilus*, and *S. costicola* subsp. *costicola*) was proposed based on polyphasic taxonomy including *16S rRNA* gene phylogeny, DNA-DNA hybridization, biochemical tests, chemotaxonomy, and genome based phylogenomic analysis [8,10]. In addition, five new species have also been identified—*S. proteolyticus* [14], *S. siamensis* [15] *S. sharmensis* [10], *S. kushneri* [9], and *S. socompensis* [7]. In recent years, 45 genomes of different *Salinivibrio* strains have been published, but most of them only provided genome statistics or were used for phylogenomic analysis [9,16]. Very little information concerning the genomic basis of survival under variable environmental conditions such as UV radiation, heavy metals, and abiotic stressors are revealed so far. This study of *Salinivibrio* sp. from the Marakkanam salt pan could provide us with a better understanding of its adaptive response and the evolution of halophilic bacteria to extreme conditions. 

The Marakkanam salt pan is subjected to large seasonal fluctuations in salinity (19–400 ppt) via evaporation, precipitation, pumping of seawater, temperature (22–34 °C), and dissolved oxygen (1.2–5 mg/L) [17]. While screening halophilic microbes from the Marakkanam salt pan, we observed that *Salinivibrio* sp. was the most dominant cultivable bacteria. We then used Illumina NextSeq sequencing in order to evaluate the genome of this species. Along with genomic analysis, we have also performed several laboratory studies to investigate the range tolerance of *Salinivibrio* sp. in response to salinity, temperature, UV radiation, and various heavy metals, which could provide insight to its adaptation strategy under extreme conditions. 

## 2. Material and Methods

### 2.1. Isolation and Identification of the Strain 

The *Salinivibrio* sp. was isolated from the Marakkanam saltpan (12°13′02′’ N; 79°58′12′’ E) in September 2017. At the time of sample collection, the salinity of the salt pan water was ~200 ppt, pH 8.1, and temperature 30 °C*. Salinivibrio* was obtained by plating the sampled water on nutrient agar prepared in the filtered and autoclaved source water. For a pure culture, the strain was subsequently streaked onto the same media. 

The isolate was identified using integrative taxonomy including morphological features, biochemical and molecular analysis, and core genome phylogeny. For phenotypic characterization, colony morphology and pigment were observed on a solid medium by stereomicroscope (Motic Electric Co. Ltd., Xiamen, China) after 24–48 h of incubation at 30 °C. To determine the basic factors for the bacterial growth such as salinity, temperature, and pH supporting the growth of bacterium, the broth cultures were incubated at different salt concentrations between 0–250 ppt, at a different temperatures from 10–50 °C, and at different pH levels from 4–10. The growth was determined by measuring the optical density at 600 nm using a UV-Vis spectrophotometer (Jasco V760, Easton, MD, USA). The cell morphology was examined by phase contrast microscopy (Nikon Instruments Inc., NY, USA). Gram staining was performed using the HiMedia commercial kit (HiMedia Laboratories, Mumbai, India). Endospore staining was performed using Schaeffer-Fulton dye (HiMedia Laboratories, Mumbai, India). Motility study was performed using semi sold media (0.8% agar, *w*/*v*) as well as by the hanging drop method on the cavity slide. The growth under anaerobic conditions was determined by incubating the bacterium in a solid medium covered with a layer of mineral oil. The catalase activity was determined by bubble production in 3% (*w*/*v*) H_2_O_2_ solution. The oxidase activity was examined using 1% (*v*/*v*) tetramethyl-p-phenylenediamine. The hydrolysis of casein, gelatin, starch, methyl red and Voges-Proskauer tests, production of indole, and Simon’s citrate and nitrate and nitrite reduction were determined according to standard procedures. The morphological and biochemical studies were conducted as per the referred protocol [18]. To determine the ability to utilize different substrates, liquid media supplemented with the test substrate was used. The acid production from carbohydrate was assessed according to Ventosa et al. [19]. For biochemical studies, the media was prepared using 60 ppt water. Unless otherwise stated, all tests were performed in triplicates and wherever required, positive and negative controls were used. The molecular identification, genomic DNA extraction, PCR amplification, and sequencing of the bacterial *16S rRNA* gene were carried out as previously described in Ravindran et al. [20]. The sequence chromatograms were analysed, edited to remove ambiguous positions, and a contig was generated using BioEdit [21]. The generated sequence was subjected to nBLAST to ascertain the identity of the organism. Sequences of the *16S rRNA* gene of related taxas were obtained from the NCBI GenBank database, and multiple sequence alignment was made using the web version of Clustal Omega (https://www.ebi.ac.uk/Tools/msa/clustalo/). The alignment was edited and a phylogenetic tree was constructed using the neighbour-Joining (NJ) method within MEGA 7.0 [22]. The sequence for the *16S rRNA* gene was submitted in the NCBI GenBank under the accession number MH071684. 

The pure culture that was designated as *Salinivibrio* sp. HTSP was maintained as glycerol stock (80% glycerol and bacterial culture, 1:1 *v*/*v*) at −80 °C for long term preservation. The strain was also deposited at the Microbial Type Culture Collection and GeneBank (MTCC), IMTECH, Chandigarh (https://mtccindia.res.in/catalog/) as *Salinivibrio* sp. under the accession number 12905.

### 2.2. IlluminaNextSeq-Based Genome Sequencing

The genomic DNA of *Salinivibrio* sp. HTSP was extracted using the method of Ravindran et al. [20]. After assuring the quality of DNA, the sequencing library was prepared using the Nextera DNA library preparation kit (Illumina Inc., San Diego, CA, USA) as per the manufacturer’s instructions. The paired-end (2 × 75 bp) sequencing was performed on the Illumina NextSeq500 (Illumina Inc.) to obtain a draft genome. The raw sequences were subjected to a quality check using FastQC (http://www.bioinformatics.babraham.ac.uk/projects/fastqc/), and trimmed using Scythe (https://github.com/vsbuffalo/scythe) and Sickle (https://github.com/najoshi/sickle). The genome was assembled on the Velvet assembler using default settings [23], and annotated automatically on the Rapid Annotations using Subsystems Technology (RAST) webserver [24]. The raw reads and assembled genome was submitted to the NCBI SRA and NCBI Genome database under the accession number SRR6847140 and PXUD00000000, respectively. The published draft genomes of other members of the genus *Salinivibrio* [9] were downloaded from NCBI GenBank and re-annotated for comparison. The details of the genome sequences used in this study are shown in Table S1. 

### 2.3. Genomic-Based Taxonomic Resolution 

The in silico DNA-DNA hybridization (DDH) was calculated by the Genome-to-Genome Distance calculator (GGDC 2.1, http://ggdc.dsmz.de/) using the BLAST+ method [25]. The results were based on recommended formula 2 (identities/HSP length), which is independent of genome length. The GGDC was also used for the genome-based species and subspecies delineation [25]. The calculation of the Average Nucleotide Identity (ANI) of the draft genome sequence used for the in silico DDH was performed in the EzBio cloud [26] as described by Yoon et al. [26], while the Ortho ANI percentage was calculated as described by Lee et al. [27]. The annotated protein-coding genes of the new isolate *HTSP* were compared with other members of *Salinivibrio* including *S. protelyticus, S. sharemsis, S. siamensis, S. costicola, S. kushneri*, three subspecies of *S. costicola,* and ten strains of *S. kushneri* using a Bacterial Pan Genome Analysis pipeline (BPGA) tool [28]. The protein fasta file of each genome, obtained as an output of RAST annotation, was used in the BPGA analysis. In the first step, BPGA compiles all the individual files into a single sequence file. This file was used for clustering with USEARCH using identity cut off 50%. The core orthologous amino acid sequences were aligned using MUSCLE, and then concatenated using the default setting of BPGA. The resulting sequence file was used for the NJ based phylogenetic reconstruction in MEGA 7.0 [22]. The functional characterization of core genes, and the unique gene of the *HTSP* strain was analysed using the COG (Cluster of Orthologous Genes) and KEGG (Kyoto Encyclopedia of Genes and Genomes) database.

### 2.4. Salinity Tolerance

To estimate the range tolerance, we streaked the strain on nutrient agar, prepared in a water source of different salinity strength (0 to 200 ppt), and incubated for 24–72 h at 30 °C. To manipulate the salinity, the salt pan water was mixed either with sterilized distilled water or salt crystals collected from the same salt pan. 

### 2.5. UV Tolerance

UV tolerance was measured by a cell survival experiment [29,30] with slight modifications. The strain was exposed to UV-A radiation (365 nm) using four TL 8W BLB 1FM/ 10X25CC bulbs (Philips Lightening Company, Chennai, TN, India) emitting wavelengths at 365 nm for up to 8 h in agar plates. 5 µL of the culture that was grown overnight with three different dilutions (10^−1^, 10^−2^, and 10^−3^), were spot inoculated on nutrient agar prepared in source water, and exposed to UV-A radiation. In total, the UV exposure experiments were conducted for 8 h, in which, after every hour duplicate plates were removed, one incubated in light and one in darkness overnight. Aluminium foil-covered glass plates were kept as the control. 

### 2.6. Heavy Metal Tolerance 

For the determination of heavy metal tolerance, an agar plating method with multiple drop inoculations were used. Nutrient Agar plates having different concentrations (0.1, 0.5, 1, 5, 10, 20, and 40 mM) of As, Ag, Cu^,^ Hg, Co, Cr, Pb, and Zn were prepared, and the overnight grown culture of *Salinivibrio* sp. HTSP was spot inoculated. Plates without heavy metals inoculated with culture were used as the control. The plates were incubated for 2–7 days, and then observed for bacterial growth. The growth was categorized as luxurious (+++), medium (++), slight (+), and no growth (-). The lowest concentration of metal, which completely prevented the growth, was considered as the minimum inhibitory concentration (MIC). Based on previously published reports, we have chosen the strain which was not inhibited by 10 mM As, 1 mM Ag, Cd, Co, Cr, Pb, Zn, and 0.1 mM of Hg, as tolerant [13,31,32]. To estimate the influence of salinity on metal tolerance, the above-described assays were repeated under different salinity conditions (30, 60, 120, 150, and 200 ppt). The required salinity was achieved as mentioned in Section 2.4. The growth was observed on plates incubated at 30 °C for 1 week.

## 3. Results

### 3.1. General Features of Salinivibrio sp. HTSP

The *Salinivibrio* sp. HTSP was isolated from the Marakkanam salt pan, Tamil Nadu (12°13′02′’ N; 79°58′12′’ E) in September 2017. Upon spreading on the solid medium, the colony appeared circular with an entire edge having a diameter of 3–4 mm, creamy, and bright coloured. The HTSP strain was Gram-negative curved rod-cells, 3–3.5 µm in length and 0.2–0.5 µm in width, motile, facultative anaerobe, and did not produce spores. Glucose and maltose were able to sustain the growth of this bacterium, while no growth was observed when lactose was used. Acid production was observed using glucose and sucrose, but not with lactose. The bacterium HTSP showed a positive result for methyl red, catalase, and oxidase, while negative for Indole, Voges-Proskauer, citrate, starch hydrolysis, and H_2_S production. The temperature range for the growth was 18–45 °C with an optimum at ~30 °C, and pH range from 5–10 with an optimum at ~7.5. The detailed description of biochemical characteristics for the HTSP strain and other closely related *Salinivibrio*s are given in Table S2.

The genome size of *Salinivibrio* sp. HTSP was found to be 3,386,080 bp. The estimated sequencing coverage depth was 100× the fold coverage and an average GC content of 50.6% was observed. The genome annotation using RAST revealed 3168 putative open reading frames (ORFs). Among them, 3150 were coding sequences (CDSs) and 18 were of RNAs (16 tRNAs and 2 rRNAs). In total, 1765 (57%) CDSs were classified according to their functions into 26 categories containing 464 subsystems using the SEED method (Figure S1). The subsystem categories with the highest number of CDS include amino acid and derivatives, carbohydrates, cofactors, and protein metabolisms. The genome of *Salinivibrio* sp. HTSP has seven confirmed and one putative CRISPR structures. Additional CDSs prediction analyses and functional annotation using NCBI COG (clusters of orthologous groups) analysis against the NCBI non-redundant protein database revealed that out of 3150 CDSs, 15.4% were assigned into amino acid transport and metabolism, 10.58% to general function, and 10.01% to translation, ribosome structure and biogenesis and so on (Figure S2). Further translated CDSs were identified for the presence of signal peptides by using the Signal P 4.1 webserver [33]. There were 235 CDSs (7.4%) containing signal peptides.

### 3.2. Taxonomic Assignment and Core Gene Phylogeny

**Figure 1:**
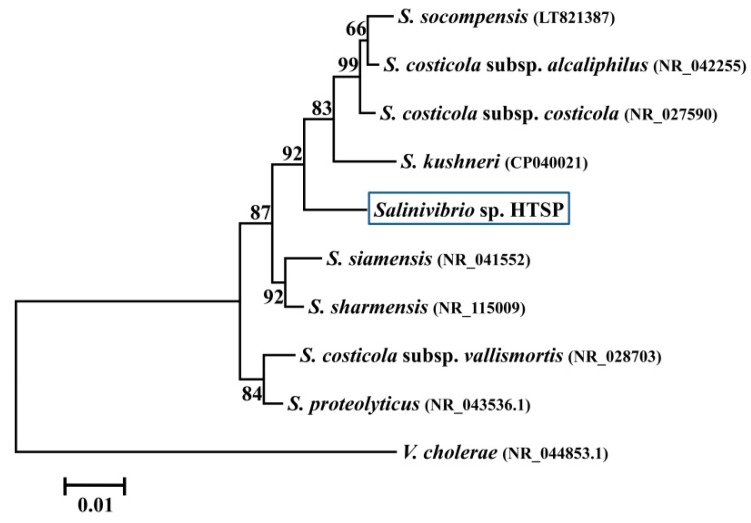
Phylogenetic tree based on *16S rRNA* gene sequences (ca.1465 bp) using the neighbour-joining method. The bootstrap test (1000 replicates) is shown next to the branches. The GenBank accession number of each sequence is shown in parenthesis. *V. cholerae* is used as an outgroup.

The almost complete *16S rRNA* (1465 bp) gene sequence analysis classified the HTSP strain in the genus, *Salinivibrio* (Figure 1). 

A closer taxonomic examination of the genome indicated that the HTSP strain had >97% Ortho Average Nucleotide Identity and >78% *in silico* Genome-to-Genome Hybridization (GGDH) with the recently described species, *S. kushneri* (Table 1).

**Figure 2:**
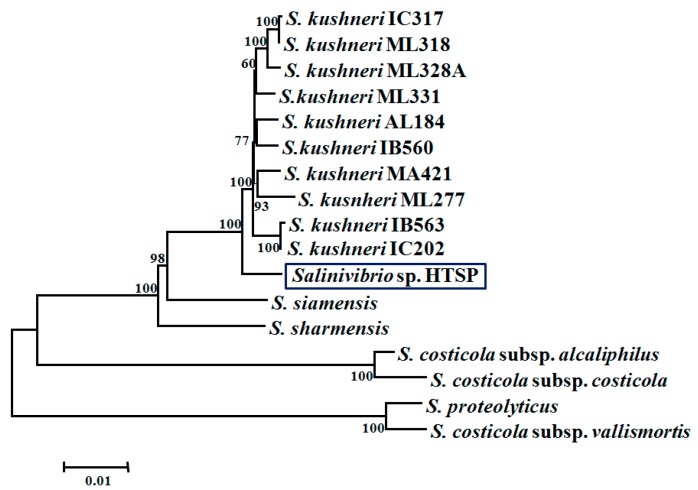
Core phylogenetic tree of *Salinivibrio* sp. HTSP and other 16 Salinvibrios sequenced by López-Hermoso et al. [9]. The tree was constructed based on the neighbour-joining distance from an alignment of 2267 core genes among all these genomes. The bootstrap test (1000 replicates) is shown next to the branch.

The BGAP analysis using USEARCH clustering indicated that the pan-genome of *HTSP* and 16 other known members of *Salinivibios* comprised of 6120 genes. Of these, 2267 genes were shared by all members, thus forming core orthologous genes. The genome of *HTSP* consists of 725 accessory genes, which were shared by at least one member of this genus, and 94 unique genes, which were not present in any other genome. Phylogenetic reconstruction based on the concatenated alignment of core genes revealed that *HTSP* forms a monophyletic group with the strains of *S. kushneri* (Figure 2). 

### 3.3. Salinity Range and Genes Related to Halotolerance

The *Salinivibrio* sp. HTSP showed a wide range of salinity tolerance (15 to 210 ppt), and it was not able to grow in the absence of salt (Table 2).

**Figure 3:**
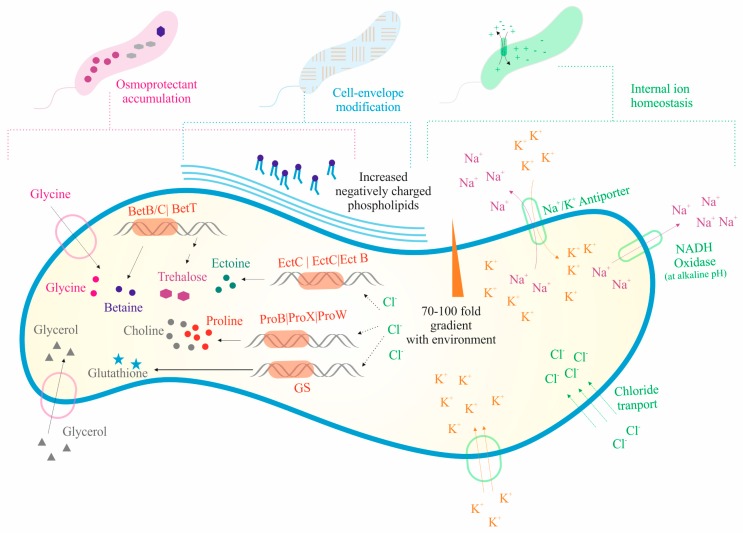
A schematic representation of salt tolerance mechanisms in *Salinivibrio* sp. HTSP based on genome analysis. Genes involved in osmoprotectant accumulation and biosynthesis such as ectoine, choline, betaine, glycerol, etc. are found in the cell. Various ion transporters such as the potassium transporter, Na^+^/H^+^ antiporter, and Cl transporters were detected for maintaining the internal ion homeostasis.

In the genome of *Salinivibrio* sp. HTSP, based on the RAST analysis with the SEED database, we identified various genes involved in the osmoregulation. We identified 22 genes involved in the biosynthesis and uptake of osmolytes such as ectoine (*di aminobutyrate-pyruvate aminotransferase*, *ectoine hydroxylase*, *l-ectoine synthase*, *l-2,4-di aminobutyric acid acetyltransferase*, *and aspartokinase)*, *choline*, *and betaine (l-proline glycine betaine ABC transport system permease protein*, *high affinity choline uptake protein*, *choline sulfatase*, *choline dehydrogenase*, *Betaine aldehyde dehydrogenase*). The complete set of osmoregulatory proU operon (*proV*, *proW*, *proX*) encoding high-affinity glycine betaine transport systems, which enable the cells to recover from the deleterious effect of hyperosmotic shock, were present. We also found one gene involved in the facilitation of glycerol uptake (*glpF*), and one involved in the synthesis of osmoregulated periplasmic glucans (*MdoB*). For internal ion homeostasis, we identified 19 genes involved in potassium transport (*TrkA*, *TrkH*, *KefB*, *KefG*, *AATP*, *FkaB*, *ATPb*, *KQT*, *FkaA, KtrA*, and *KtrB*), one for the H^+^/Cl^−^ ClcA transporter, and seven for the Na^+^/H^+^ antiporter. The details of the genomic features associated with the osmotic stress response are given in Table S3. Additionally, the genome also contained a large number of CDS for metabolisms and transporters such as amino acid transporters and ABC transporters, with osmotic stress-related functions (Figure 3).

### 3.4. Resistance to UV-A Radiation 

**Figure 4:**
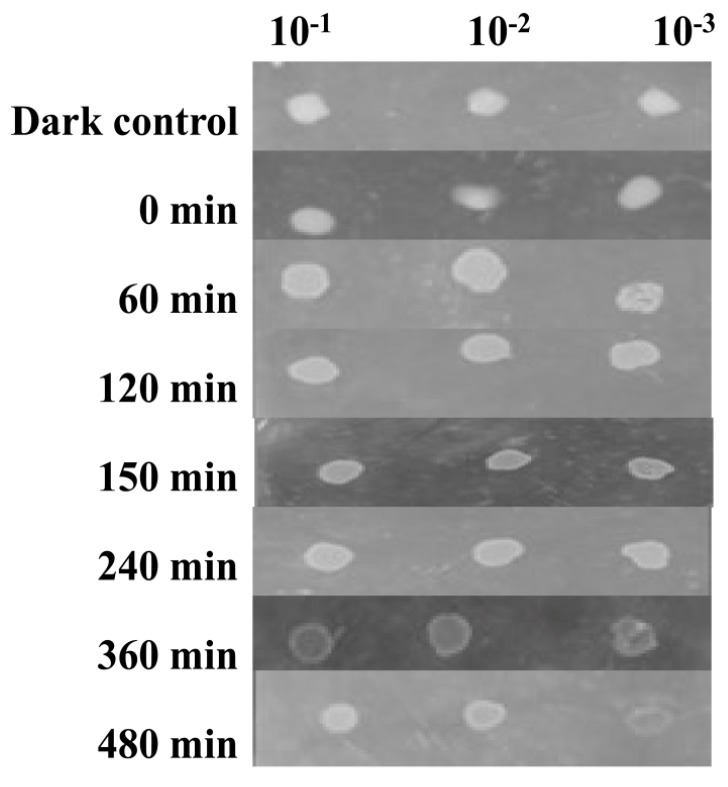
Screening of resistance to UV radiation: Growth and survival of *Salinivibrio* sp. HTSP monitored after exposing to UV for different time points.

*Salinivibrio* sp. HTSP was able to resist a UV-A radiation of 365 nm for ~8 h. The serially diluted (10^−1^, 10^−2^, and 10^−3^) drops of culture were grown within 1 h upon incubation in both dark and light conditions after UV-A exposure (Figure 4).

**Figure 5:**
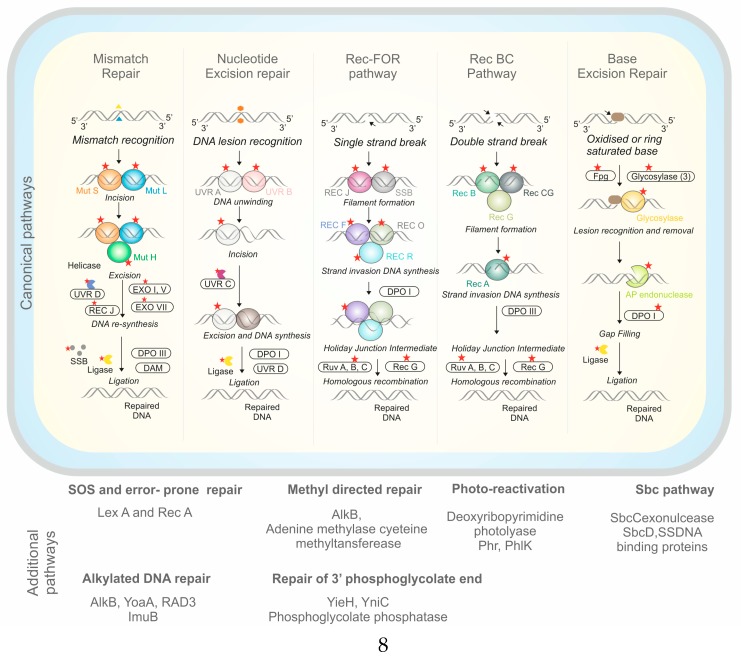
DNA repair genes and pathways putatively responsible for UV tolerance *in Salinivibrio* sp. HTSP based on genome analysis. The red star indicates the presence of gene in each pathway stands for the possible mechanism of DNA repair.

UV resistance is a complex process that includes a diversity of DNA repair mechanisms. In the genome of the HTSP strain, we found 72 genes involved in the various DNA repair pathways. We found a complete set of genes encoding enzymes for excision repair, mismatch repair, and recombinational repair (Figure 5).

For base excision repair, 10 genes were identified which included *glycosylase*, *AP endonuclease, DNA polymerase, and Ligase*. A total of seven genes involved in nucleotide excision repair were identified including *Uvr A*, *Uvr B*, *Uvr C nuclease*, and *Uvr D helicase.* For the mismatch repair system, nine genes for *MutS, MutH, DNA polymerase, helicases II (Uvr D), exonuclease (IV, V and Rec J)*, *single-strand binding protein (SSB)*, and *ligase* were identified. In the Rec BCD, Rec FOR, and SbcBCD pathways, 34 genes were identified for *Rec A*, *Rec B*, *Rec C*, *Rec D*, *Rec F*, *Rec O*, *Rec R*, *Rec J*, *SSB*, *SbcC*, *SbcD*, *RuvA*, *RuvB*, *RuvC*, and *RecG*. We also found two genes for *deoxy-ribo-dipyrimidine-photolyase*, involved in direct photoreactivation, one gene each for *Lux A* and *Rec A* in SOS response, one gene each for *phospho-glycolate-phosphatase* and *2-deoxyglucose-6-phosphate hydrolase* in phosphoglycolate salvage, one gene each for methyl-directed repair *DNA adenine methylase* and *methylated-DNA-protein-cysteine methyl transferase* in methyl directed, two genes each for *Alk B* and *Imu C* in alkylated DNA repair, and one gene each for *DinG*, *Rad3*, and *Yoa A* for ATP-dependent DNA polymerase (Figure 5).

### 3.5. Heavy Metal Tolerance

Based on the MIC assay, at the optimum salinity of 12% NaCl, the HTSP strain showed tolerance against six heavy metals including Cu, Zn, Co, Hg Cr, and Pb, showed a resistance to As, and was sensitive to Ag. In general, the salinity showed a marked influence on heavy metal tolerance (Table 3). The tolerance level to As, Cu, Co, and Pb increased, whereas it decreased for Zn and Cr under higher salinity conditions (Table 3).

**Figure 6:**
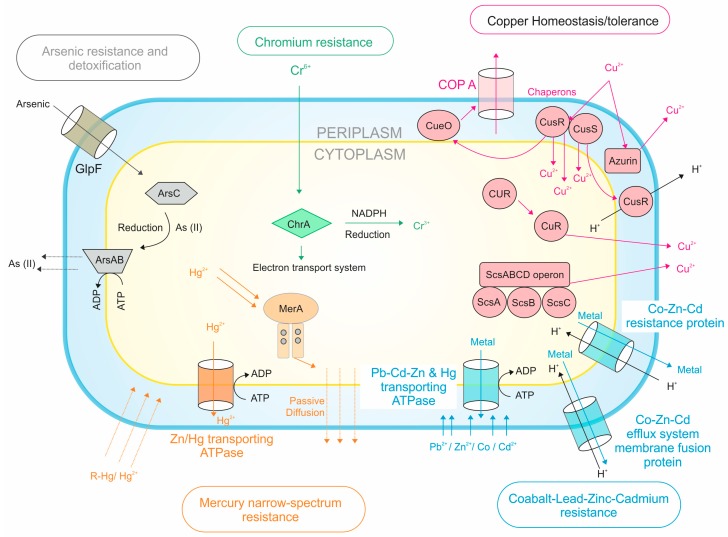
An overview for possible genes and pathways involved in heavy metal resistance in *Salinivibrio* sp. HTSP based on genome analysis.

Upon genome analysis, we found 20 genes for copper homeostasis/tolerance (four different mechanisms e.g., cytoplasmic Cue system (*CoPA* and *CueO*), Cus system (*CusR* and *CusS*), Copper activated operon ScsABCD (*ScsA*, *ScsB*, *ScsC*, and *ScsD*), and periplasmic CopA system (*CoPA*, *CueO, CopG*, and *CcoA*), 12 genes for Co, Zn, Cd, and Pb tolerance (three different efflux transporters e.g., *P-type ATPase*, *CBA transporter*, *and CDF transporters*), three genes for Hg tolerance (*translocating ATPase and Mercuric reductase*), seven genes for As resistance (three different mechanisms e.g., one gene for arsenate reductase capable of reducing arsenate, one gene for arsenical-resistance protein Acr3 which is involved in the extrusion of arsenate from cells, and five genes involved in arsenate and arsenite resistance operon (arsRBHC)), and one gene for chromium resistance (*Chromate transporter protein*). All the possible genomic mechanisms underlying heavy metal resistance in *Salinivibrio* sp. has been given in Figure 6.

### 3.6. General Stress-Related Genes

In total, 97 genes encoding for proteins involved in protection against various abiotic stress conditions were identified. The genome had eight genes involved in the periplasmic stress response (*DegS*, *RseP*, *RseA*, *Skp*, *SurA*, *DegQ*, and *RseB*) for sensing stress under changing environments. To combat oxidative stress, the strain had 48 genes, including four for superoxide dismutase (SOD), three for catalase, two for peroxidase, four for glutathione synthetase (GS), seven for glutathione-s-transferase, and 41 other genes including carbon starvation (5), Cold shock (3), and heat shock (16) were also identified. The details of the genes are presented in Table S4.

## 4. Discussion

Halophilic bacteria are increasingly being employed as a source for secondary metabolites with biotechnological applications [35]. Recently, they have also been seen as suitable in bioremediation and biomonitoring of sites polluted with heavy metals. In the present study, we have isolated a halophilic bacterial strain from the Marakkanam salt pan and identified it as *S. kushneri* based on polyphasic taxonomy. However, the strain differed in some biochemical tests such as sucrose utilization, methyl red test, Voges Prauskauer, and lactose fermentation, from a closely related strain of *S. kushneri* reported from solar salterns in Spain [34]. In the present study, we have analysed the genome of *Salinivibrio* sp. HTSP in order to understand the base of its adaptation under variable environmental conditions as well as to determine the role of this species in the transformation and bioremediation of heavy metal polluted sites in nature.

### 4.1. Strategies for Halo Adaptation

*Salinivibrio* had different strategies to adapt with variable salinity concentrations like accumulation of osmoprotectants such as sugars, polyols, and amino acids and its derivatives [36]; modification of the cell envelope phospholipids, maintenance of the internal ion concentration, and ion pumps on the cell membrane [37]; and the ability to control water movement in and out and maintain a hypo-osmotic state of their intracellular space [11]. 

The intracellular environment of *Salinivibrios* contains compatible osmolytes, and they provide an osmotic balance without interfering with the metabolic function of the cells. These compounds include zwitterionic solutes such as glycine, betaine, and ectoine; uncharged solutes such as trehalose; and anionic solutes such as glutamate, and hydroxyl butyrate [38]. In the salt-sensitive mutant of *S. costicola* subsp. *yaniae*, the accumulation of glycine, betaine, and ectoine was observed upon salinity exposure [36]. In the study, it was also found that glycine and betaine were taken up from the medium, and glutamate and ectoine carried out an important role for the cell growth in a saline environment. The presence of the *BetB, BetL, BetT, EctA, EctB,* and *EctC* genes indicate that *Salinivibrios* have the potential for synthesizing these compatible osmolytes under osmotic stress, and the presence of the glycerol uptake facilitator (*GlpF*) indicates the use of glycerol asosmolytes under osmotic stress conditions [11,39]. The cells achieve a low intracellular ionic environment in comparison to the extracellular environment by transporting ions using the Na^+^/H^+^-antiporter [40], having primary respiration-driven Na^+^ pumps [41], and having potassium transport and efflux pumps [42]. The genes such as *TrkA* and TrkH are responsible for the uptake of potassium; *Kqt, Kef B,* and *Kef G* for potassium efflux; *AATP, FkaB, ATPb, FkaA, KtrA,* and *KtrB* for potassium homeostasis; and *H^+^/Cl^-^ClcA* and *Na^+^/H^+^ antiporter* for the transport of sodium ions [39,42]. Previous studies have also reported that the membrane phospholipids and the type of fatty acid chains change upon sensing variable salt concentrations in the growth medium [43]. 

### 4.2. Genomic Basis of UV Tolerance 

UV radiation is harmful to microorganisms due to their small, haploid genomes and lack of thick protective cell walls [44]. A great diversity of microbes under extreme conditions may possess diverse mechanisms to resist the harmful radiations [45]. UV-A is most common one, and comprises 95% of the total UV energy that reaches the Earth’s surface [46]. UV-A radiation penetrates more profoundly into the saline water than other mediums [47]. The salt in the surrounding environment causes mobilization of atmospheric chlorine, leading to more UV exposure [48]. UV-A acts by producing photo-oxidizing compounds and ROS that can damage biomolecules such as DNA, proteins, and lipids [49].

The molecular mechanism behind UV radiation resistance in bacteria and archaea mainly comes into the light repair mechanism such as photoreactivation, and dark repair mechanism such as excision repair, mismatch repair, and recombination repair, in addition to the SOS response [1,30]. During photoreactivation, the genes such as *deoxy-ribo-di-pyrimidine photolyase (phr)*, a protein related to *deoxy-ribo-di-pyrimidine photolyase (phlK)* that utilizes visible light, and reverses UV-induced lesions and photoproducts by directly rearranging bonds [50]. Similar to the HTSP strain in the present study, *phr* and *phlK* were also reported in different strains of *Acinetobacter* sp. [29,30]. *G*ene knockout studies of phr genes in halophilic archaeon *Halobacterium* sp. also supported the role of these genes in the strong photo repair activity and efficient CPD repair [51,52].

Nucleotide excision repair is a multistep process in bacteria that requires identification of the spot of damage, slicing of the damaged strand, removal of the damaged strand, and synthesis and joining of new strands. The genes generally involved in this process are the *uvrABCD, DNA polymerases, ligases* [53,54], and *DNA glycosylase* [55]. The double-strand breaks and lesions in DNA due to UV radiation are healed by recombinational repair through DSB recognition, excision, binding of the enzyme, branch migration, and finally branch resolution [56]. The pathways such as RecBCD, RecFOR, and SbcBCD, and its associated genes such as *Rec A, Rec B, RecC*, and *Rec D* were involved in this repair [39,53]. Similar to *Salinivibrio* sp., a complete set of genes and DNA repair pathways were previously reported in several other halophilic bacteria from different extreme conditions including *Acinetobacter* sp., *Exiguobacterium* sp., *and Stenotrophomonas maltophila* [3,30,45].

In *Salinivibrio* sp. HTSP, we also observed a few unique genes for DNA repair, which were not yet reported in other *Salinivibrios.* These genes included *RuvA, RuvB, RuvC,* and *Rec G,* which were involved in the RecFOR and SbcBCD pathways as well as *PhlK* of photoreactivation [30], *RadA* of recombinational repair [57], *RadC* of DNA double-strand repair, and *Rec X* of SOS repair [58]. 

### 4.3. Heavy Metal Tolerance 

The bacterial strain could achieve heavy metal tolerance through different mechanisms like metal sequestration, metal-specific efflux pumps, and enzymatic detoxification [59]. Physiological and genomic adjustments play a key role in maintaining homeostasis under stressful conditions [60]. In the genome of *Salinivibrio* sp. HTSP, we observed genes for the Cue system that indicated *COPA*, a copper translocating ATPase and a multicopper oxidase *(CueO)* which act together as an efflux pump in order to pump out excess Cu^2+^ from the cell [61,62]. The presence of CUS (copper sensing locus) may help bacterial cells to export metal ions outside the cell in exchange for the influx of H^+^ [63]. Copper-activated operon such as ScsABCD (*ScsA, ScsB, ScsC,* and *ScsD*) together with copper-inducible promoters such as CpxR/CpxA and periplasmic copper-accepting protein azurin seems to further contribute toward copper tolerance in *Salinivibrio* sp. HTSP. The copper tolerance has been described in different species of halophilic bacteria [13,31,32]. Analysis of the genomes of 268 gamma Proteobacteria gave insights into 1417 orthologs of different copper homeostasis genes, including 85% for *CopA*, 77% for *CusC*, 60% for *CusA*, 53% for *CusB*, 37% for *CueO*, and 36% for *CutF* [46].

Arsenic tolerance in bacteria is mediated through three mechanisms such as arsenic reduction, extrusion of arsenate, and via arsenite resistance operon [64]. Arsenate and arsenite resistance operon (arsRBHC) plays a major role in arsenic resistance. In this detoxification system, the cytoplasmic oxidoreductase, *ArsC*, reduces arsenate to arsenite, and the toxic arsenite is excreted through the ArsAB efflux pump [39,64]. Similar mechanisms of arsenic tolerance has been reported in other halobacteria such as *Halobacterium* sp., *E. coli*, and *Staphylococcus aureus,* etc. [65,66].

Bacteria acquire Co, Zn, Cd, and Pb tolerance through major three different classes of efflux transporters such as P-type ATPase, CBA transporter, and CDF transporters [67,68]. Among these, P-type ATPase pumps out metal ion from the cytoplasm to periplasm [68]. The presence of CBA transporters contributes to the high level of resistance against metal by pumping metal ions out of the cells [68]. In the genome, we have found genes involved in the Co/Zn/Cd efflux system, membrane fusion protein, and the CDF transporter (Cobalt-zinc-cadmium resistance protein), which detoxifies the periplasmic metal ions [68]. Similar to *Salinivibrio* sp., these genes were also reported in several other bacteria including *Arthrobacter spp*., *Bacillus megaterium*, *Pseudomonas marginalis*, *Citrobacter freundii*, *Staphylococcus aureus, H. xingjiangensis*, *Chromohalobacter israelensis,* and *Salinicola socius* [69,70]. For mercury resistance, mercury-translocating ATPase transports the excess mercury, and other enzymes such as the mercury reductase, *Mer A*, reduces the toxic mercury [71]. The genes of mercury resistance are found in *mer* operons, and *Mer A* is considered as a central enzyme in bacteria which catalyzes the reduction of mercury [72]. Genomic analyses revealed the presence of the mercury resistance system in metal-tolerant halophiles such as *C. israelensis* and *H. zicindurans* [73] as well as in halotolerant bacteria such as *Idiomarina loihiensis* and halophilic bacteria such as *Halothiobacillus neapolitanus* [74]. Chromate efflux is mediated by the *ChrA* gene and it is the key mechanism of chromate detoxification [75,76]. Similar to the *Salinivibrio* sp. HTSP genome, the presence of the chromium resistance operon was also identified in *Ochrobactrum tritici* [77]. 

### 4.4. General Stress-Responsive Genes

In *Salinivibrio sp*. the genome analysis revealed that there were at least 97 genes associated with the general stress response, which is broadly categorized into periplasmic stress response and oxidative stress response, and are discussed separately below.

#### 4.4.1. Periplasmic Stress Response

Transcriptional control is a common form of stress response in the bacteria [78]. In *Salinivibrio* sp., periplasmic stress genes such as *DegS*, *RseP*, *RseA*, *Skp*, *SurA*, *DegQ*, *RseB*, *CpxR*, and *CpxA* may be contributing to the bacteria’s ability to survive physiologically stressful conditions by responding to the misfolding of outer membrane proteins [79], or by initiating transcription through the release of a sigma e subunit of RNA polymerase [80], and by stabilizing regulatory proteins [81].

Besides this system, the Cpx two-component transcriptional systems help bacterial cells survive the pH of stress conditions. The Cpx system senses misfolded inner membrane proteins through modifications in the phosphoryl transfer reactions between the membrane-bound sensor, *histidine kinase CpxA,* and the cytoplasmic response regulator *CpxR*, and it allows the bacteria to survive [80]. The bearing of such genes makes it evident that *Salinivibrio sp*. HTSP may be using similar mechanisms to adapt in the harsh conditions of the Marakkanam salt pan.

#### 4.4.2. Oxidative Stress Response

Exposure to environmental stress such as UV radiation, desiccation, pH, and elevated temperature can damage cells directly through oxidative stress [82]. Oxidative stress varies among bacteria, however it mostly affects the different cellular processes [83] including damage to cell wall components, membrane lipids, nucleic acids, and proteins [84]. There are different strategies to cope with ROS accumulation, in which enzymatic actions by catalases and superoxide dismutase (SOD) plays a vital role [85]. Genomic studies on *Salinivibrio* sp. revealed the presence of catalase, peroxidase, and SOD. SOD forms the first line of defense against ROS as it converts free radicals into lesser toxic H_2_O_2_, then other enzymes such as catalase, glutathione peroxidase, and glutathione-*S*-transferase neutralize H_2_O_2_ to water and organic peroxide [85,86]. 

Heat shock proteins (HSPs) are another important protein expressed in response to environmental stimuli including abiotic and biotic stressors [87]. Previous works have demonstrated a close relationship between inductions of these HSPs with increased tolerance of higher temperatures [88]. In *Salinivibrio* sp., we have identified multiple copies of different classes of HSPs, which may help the strain cope with the high temperature variations in salt pans. 

Glutathione and related gene families help the bacteria in maintaining a static oxidation state, prevents the cell from the action of low pH, chlorine compounds, and plays a key role in direct protein modification under stress [89]. Likewise, the genes of carbon starvation (Starvation lipoprotein *Slp*) protect the bacterial cells from the hydrogen peroxide stress as well as heat and osmotic conditions by synthesizing catalase [90,91].

## 5. Conclusion

The genomic and phenotypic attributes of *Salinivibrio* sp. HTSP revealed the ability to adapt under a wide range of salinities, heavy metals, and under higher UV radiation. Several unique genes were also identified in comparison with other available genomes. In the future, genomic information obtained in the present study can be used to gain a deeper understanding of the regulation of metabolic pathways under stressful conditions. Further, this strain can also be used in different applications such as UV absorbance and bioremediation, etc.

## Reference

## Figures and Tables

**Table 1:** *In silico* Genome-to-Genome Hybridization (GGDH), and OrhtoANI values of *Salinivibrio* sp. HTSP with 10 other strains of *Salinivibrio kushneri* isolated from the salterns in Spain [34].

	GGDH	OrthoANI
*S. kushneri IB560*	79.90%	97.79%
*S. kushneri IB563*	78.10%	97.61%
*S. kushneri ML338A*	80.50%	97.79%
*S. kushneri IC317*	80.30%	97.84%
*S. kushneri MA421*	78.40%	97.62%
*S. kushneri ML318*	80.40%	97.94%
*S. kushneri AL184*	81.10%	97.79%
*S. kushneri ML331*	80.10%	97.76%
*S. kushneri IC202*	78%	97.64%
*S. kushneri ML277*	79.90%	97.75%

**Table 2:** Salinity tolerance of *Salinivibrio* sp. HTSP.

Salinity (ppt)	Growth	Time Taken for Growth in Days at ~30 °C
0	Absent	
15	Present	Overnight incubation
30	Present	Overnight incubation
60	Present	Overnight incubation
120	Present	2 days
150	Present	2 days
200	Present	3 days

**Table 3:** Heavy metal tolerance of *Salinivibrio sp*. under different salinity.

Salinity	30 ppt			60 ppt			120 ppt			150 ppt			200 ppt		
Concentrations	1 mM	5 mM	10 mM	1 mM	5 mM	10 mM	1 mM	5 mM	10 mM	1 mM	5 mM	10 mM	1 mM	5 mM	10 mM
Arsenic	+++	+++	-	+++	+++	-	+++	+++	-	+++	+++	+	+++	+++	+
Copper	+++	+	-	+++	+	-	+++	++	-	+++	+++	+	+++	+++	+
Cobalt	+++	+++	+	+++	+++	++	+++	+++	++	+++	+++	++	+++	+++	++
Zinc	+++	+++	++	+++	+++	++	+++	+	-	+++	-	-	+++	-	-
Chromium	+++	-	-	+++	-	-	+++	-	-	+++	-	-	++	-	-
Lead	+++	+++	+++	+++	+++	+++	+++	+++	+++	+++	+++	+++	+++	+++	+++

The growth is categorized as luxurious (+++), medium (++), slight (+), and no growth (-) based on visual observation.

## Supplementary Materials

The following are available online at https://www.mdpi.com/2076-2607/7/11/577/s1.

## References

[1] Jones D.L., Baxter B.K. (2017). DNA repair and photoprotection: Mechanisms of overcoming environmental ultraviolet radiation exposure in halophilic archaea. Front. Microbiol..

[2] Agogué H., Joux F., Obernosterer I., Lebaron P. (2005). Resistance of marine bacterioneuston to solar radiation. Appl. Environ. Microbiol..

[3] Flores M.R., Ordoñez O.F., Maldonado M.J., Farías M.E. (2009). Isolation of UV-B resistant bacteria from two high altitude Andean lakes (4400 m) with saline and non saline conditions. J. Gen. Appl Microbiol..

[4] Krishna P.S., Sreenivas A., Singh D.K., Shivaji S., Prakash J.S. (2015). Draft genome sequence of *Bacillus okhensis* Kh10-101T, a halo-alkali tolerant bacterium from Indian salt pan. Genom. Data.

[5] Paraneeiswaran A., Shukla S.K., Prashanth K., Rao T.S. (2015). Microbial reduction of [Co (III)–EDTA]− by *Bacillus licheniformis* SPB-2 strain isolated from a solar salt pan. J. Hazard. Mater..

[6] Dey R., Pal K.K., Sherathia D., Dalsania T., Savsani K., Patel I., Sukhadiya B., Mandaliya M., Thomas M., Ghorai S. (2013). Draft genome sequence of *Bacillus* sp. strain NSP2. 1, a nonhalophilic bacterium isolated from the salt marsh of the Great Rann of Kutch, India. Genome Announc..

[7] Galisteo C., Sánchez-Porro C., de la Haba R.R., López-Hermoso C., Fernández A.B., Farias M.E., Ventosa A. (2019). Characterization of *Salinivibrio socompensis* sp. *nov.*, A New Halophilic Bacterium Isolated from the High-Altitude Hypersaline Lake Socompa, Argentina. Microorganisms.

[8] Huang C.-Y., Garcia J.-L., Patel B., Cayol J.-L., Baresi L., Mah R.A. (2000). *Salinivibrio costicola* subsp. *vallismortis* subsp. nov., a halotolerant facultative anaerobe from Death Valley, and emended description of *Salinivibrio costicola*. Int. J. Syst. Evol. Microbiol..

[9] López-Hermoso C., Rafael R., Sánchez-Porro C., Bayliss S.C., Feil E.J., Ventosa A. (2017). Draft Genome Sequences of *Salinivibrio proteolyticus, Salinivibrio sharmensis, Salinivibrio siamensis, Salinivibrio costicola subsp. alcaliphilus, Salinivibrio costicola subsp. vallismortis*, and 29 New Isolates Belonging to the Genus Salinivibrio. Genome Announc..

[10] Romano I., Orlando P., Gambacorta A., Nicolaus B., Dipasquale L., Pascual J., Giordano A., Lama L. (2011). *Salinivibrio sharmensis* sp. nov., a novel haloalkaliphilic bacterium from a saline lake in Ras Mohammed Park (Egypt). Extremophiles.

[11] Ventosa A., Nieto J.J., Oren A. (1998). Biology of moderately halophilic aerobic bacteria. Microbiol. Mol. Biol. Rev..

[12] Mellado E., Moore E., Nieto J., Ventosa A. (1996). Analysis of *16S rRNA* gene sequences of *Vibrio costicola* strains: Description of *Salinivibrio costicola* gen. nov., comb. nov.. Int. J. Syst. Evol. Microbiol..

[13] Garcia M., Nieto J., Ventosa A., Ruiz-Berraquero F. (1987). The susceptibility of the moderate halophile Vibrio costicola to heavy metals. J. Appl. Bacteriol..

[14] Amoozegar M.A., Schumann P., Hajighasemi M., Fatemi A.Z., Karbalaei-Heidari H.R. (2008). *Salinivibrio proteolyticus* sp. nov., a moderately halophilic and proteolytic species from a hypersaline lake in Iran. Int. J. Syst. Evol. Microbiol..

[15] Chamroensaksri N., Tanasupawat S., Akaracharanya A., Visessanguan W., Kudo T., Itoh T. (2009). *Salinivibrio siamensis* sp. nov., from fermented fish (pla-ra) in Thailand. Int. J. Syst. Evol. Microbiol..

[16] López-Hermoso C., de la Haba R.R., Sánchez-Porro C., Papke R.T., Ventosa A. (2017b). Assessment of MultiLocus Sequence Analysis as a valuable tool for the classification of the genus *Salinivibrio*. Front. Microbiol..

[17] Kabilan M. (2016). quot Microbial diversity of halophilic archaea and bacteria in solar salterns and studies on their production of antiarchaeal substancesquot. Ph.D. thesis.

[18] Prescott L.M., Sherwood L., Woolverton C. (2007). Prescott, Harley, and Klein’s Microbiology.

[19] Ventosa A., Quesada E., Rodriguez-Valera F., Ruiz-Berraquero F., Ramos-Cormenzana A. (1982). Numerical taxonomy of moderately halophilic Gram-negative rods. Microbiology.

[20] Ravindran J., Kannapiran E., Manikandan B., Francis K., Arora S., Karunya E., Kumar A., Singh S., Jose J. (2013). UV-absorbing bacteria in coral mucus and their response to simulated temperature elevations. Coral Reefs.

[21] Hall T.A. (1999). BioEdit: A user-friendly biological sequence alignment editor and analysis program for Windows 95/98/NT. Nucleic Acids Symposium Series.

[22] Kumar S., Stecher G., Tamura K. (2016). MEGA7: Molecular evolutionary genetics analysis version 7.0 for bigger datasets. Mol. Biol. Evol..

[23] Zerbino D.R., Birney E. (2008). Velvet: Algorithms for de novo short read assembly using de Bruijn graphs. Genome Res..

[24] Overbeek R., Olson R., Pusch G.D., Olsen G.J., Davis J.J., Disz T., Edwards R.A., Gerdes S., Parrello B., Shukla M. (2013). The SEED and the Rapid Annotation of microbial genomes using Subsystems Technology (RAST). Nucleic Acids Res..

[25] Meier-Kolthoff J.P., Auch A.F., Klenk H.-P., Göker M. (2013). Genome sequence-based species delimitation with confidence intervals and improved distance functions. BMC Bioinform..

[26] Yoon S.-H., Ha S.-m., Lim J., Kwon S., Chun J. (2017). A large-scale evaluation of algorithms to calculate average nucleotide identity. Antonie Van Leeuwenhoek.

[27] Lee I., Kim Y.O., Park S.-C., Chun J. (2016). OrthoANI: An improved algorithm and software for calculating average nucleotide identity. Int. J. Syst. Evol. Microbiol..

[28] Chaudhari N.M., Gupta V.K., Dutta C. (2016). BPGA-an ultra-fast pan-genome analysis pipeline. Sci. Rep..

[29] Albarracín V.H., Pathak G.P., Douki T., Cadet J., Borsarelli C.D., Gärtner W., Farias M.E. (2012). Extremophilic *Acinetobacter* strains from high-altitude lakes in Argentinean Puna: Remarkable UV-B resistance and efficient DNA damage repair. Orig. Life Evol. Biosph..

[30] Kurth D., Belfiore C., Gorriti M.F., Cortez N., Farias M.E., Albarracín V.H. (2015). Genomic and proteomic evidences unravel the UV-resistome of the poly-extremophile *Acinetobacter sp.* Ver3. Front. Microbiol..

[31] Nieto J., Fernandez-Castillo R., Marquez M., Ventosa A., Quesada E., Ruiz-Berraquero F. (1989). Survey of metal tolerance in moderately halophilic eubacteria. Appl. Environ. Microbiol..

[32] Nieto J., Ventosa A., Ruiz-Berraquero F. (1987). Susceptibility of halobacteria to heavy metals. Appl. Environ. Microbiol..

[33] Petersen T.N., Brunak S., Von Heijne G., Nielsen H. (2011). SignalP 4.0: Discriminating signal peptides from transmembrane regions. Nat. Methods.

[34] López-Hermoso C., Rafael R., Sánchez-Porro C., Ventosa A. (2018). *Salinivibrio kushneri* sp. nov., a moderately halophilic bacterium isolated from salterns. Syst. Appl. Microbiol..

[35] Selvarajan R., Sibanda T., Tekere M., Nyoni H., Meddows-Taylor S. (2017). Diversity analysis and bioresource characterization of halophilic bacteria isolated from a South African salt pan. Molecules.

[36] Zhu D., Zhang W., Zhang Q., Nagata S. (2010). Accumulation and role of compatible solutes in fast-growing *Salinivibrio costicola* subsp. *yaniae*. Can. J. Microbiol..

[37] Gunde-Cimerman N., Plemenitaš A., Oren A. (2018). Strategies of adaptation of microorganisms of the three domains of life to high salt concentrations. Fems Microbiol. Rev..

[38] Galinski E.A. (1993). Compatible solutes of halophilic eubacteria: Molecular principles, water-solute interaction, stress protection. Experientia.

[39] Gorriti M.F., Dias G.M., Chimetto L.A., Trindade-Silva A.E., Silva B.S., Mesquita M.M., Gregoracci G.B., Farias M.E., Thompson C.C., Thompson F.L. (2014). Genomic and phenotypic attributes of novel *salinivibrios* from stromatolites, sediment and water from a high altitude lake. BMC Genom..

[40] Udagawa T., Unemoto T., Tokuda H. (1986). Generation of Na+ electrochemical potential by the Na+-motive NADH oxidase and Na+/H+ antiport system of a moderately halophilic *Vibrio costicola*. J. Biol. Chem..

[41] Hamaide F., Kushner D.J., Sprott G.D. (1983). Proton motive force and Na+/H+ antiport in a moderate halophile. J. Bacteriol..

[42] Epstein W. (2003). The roles and regulation of potassium in bacteria. Prog. Nucleic Acid Res. Mol. Biol..

[43] Russell N., Kogut M., Kates M. (1985). Phospholipid Biosynthesis in the Moderately Halophilic Bacterium *Vibvio costicola* During Adaptation to Changing Salt Concentrations. Microbiology.

[44] Ponder M.A., Gilmour S.J., Bergholz P.W., Mindock C.A., Hollingsworth R., Thomashow M.F., Tiedje J.M. (2005). Characterization of potential stress responses in ancient Siberian permafrost psychroactive bacteria. FEMS Microbiol. Ecol..

[45] Ordoñez O.F., Flores M.R., Dib J.R., Paz A., Farías M.E. (2009). Extremophile culture collection from Andean lakes: Extreme pristine environments that host a wide diversity of microorganisms with tolerance to UV radiation. Microb. Ecol..

[46] Hernández-Montes G., Argüello J.M., Valderrama B. (2012). Evolution and diversity of periplasmic proteins involved in copper homeostasis in gamma proteobacteria. BMC Microbiol..

[47] Huovinen P., Penttilä H., Soimasuo M. (2003). Spectral attenuation of solar ultraviolet radiation in humic lakes in Central Finland. Chemosphere.

[48] Stutz J., Ackermann R., Fast J.D., Barrie L. (2002). Atmospheric reactive chlorine and bromine at the Great Salt Lake, Utah. Geophys. Res. Lett..

[49] Wilson C., Caton T., Buchheim J., Buchheim M., Schneegurt M., Miller R. (2004). DNA-repair potential of *Halomonas spp*. from the Salt Plains Microbial Observatory of Oklahoma. Microb. Ecol..

[50] Sancar G.B. (2000). Enzymatic photoreactivation: 50 years and counting. Mutat. Res. Fund. Mol. Mech..

[51] Baliga N.S., Bjork S.J., Bonneau R., Pan M., Iloanusi C., Kottemann M.C., Hood L., DiRuggiero J. (2004). Systems level insights into the stress response to UV radiation in the halophilic archaeon *Halobacterium* NRC-1. Genome Res..

[52] McCready S., Marcello L. (2003). Repair of UV damage in *Halobacterium salinarum*. Biochem. Soc. Trans..

[53] Capes M.D., Coker J.A., Gessler R., Grinblat-Huse V., DasSarma S.L., Jacob C.G., Kim J.-M., DasSarma P., DasSarma S. (2011). The information transfer system of halophilic archaea. Plasmid.

[54] Zhao A., Gray F.C., MacNeill S.A. (2006). ATP-and NAD+-dependent DNA ligases share an essential function in the halophilic archaeon *Haloferax volcanii*. Mol. Microbiol..

[55] Krokan H.E., Bjørås M. (2013). Base excision repair. Cold Spring Harb. Perspect. Biol..

[56] Cox M.M. (1991). The RecA protein as a recombinational repair system. Mol. Microbiol..

[57] Zhou Q., Zhang X., Xu H., Xu B., Hua Y. (2006). RadA: A protein involved in DNA damage repair processes of *Deinococcus radiodurans* R1. Chin. Sci. Bull..

[58] Pagès V., Koffel-Schwartz N., Fuchs R.P. (2003). recX, a new SOS gene that is co-transcribed with the recA gene in *Escherichia coli*. DNA Repair.

[59] Nies D.H. (1999). Microbial heavy-metal resistance. Appl. Microbiol. Biotechnol..

[60] Kaur A., Pan M., Meislin M., Facciotti M.T., El-Gewely R., Baliga N.S. (2006). A systems view of haloarchaeal strategies to withstand stress from transition metals. Genome Res..

[61] Das D., Salgaonkar B.B., Mani K., Braganca J.M. (2014). Cadmium resistance in extremely halophilic archaeon *Haloferax* strain BBK2. Chemosphere.

[62] Ladomersky E., Petris M.J. (2015). Copper tolerance and virulence in bacteria. Metallomics.

[63] Munson G.P., Lam D.L., Outten F.W., O’Halloran T.V. (2000). Identification of a copper-responsive two-component system on the chromosome of *Escherichia coli* K-12. J. Bacteriol..

[64] Rosen B.P. (2002). Transport and detoxification systems for transition metals, heavy metals and metalloids in eukaryotic and prokaryotic microbes. Comp. Biochem. Physiol. A Mol. Integr. Physiol..

[65] Lin Y.-F., Walmsley A.R., Rosen B.P. (2006). An arsenic metallochaperone for an arsenic detoxification pump. Proc. Natl. Acad. Sci. USA.

[66] Wang H., Li H., Shao Z., Liao S., Johnstone L., Rensing C., Wang G. (2012). Genome sequence of deep-sea manganese-oxidizing bacterium *Marinobacter manganoxydans* MnI7-9. Am. Soc. Microbiol..

[67] Hynninen A. (2010). Zinc, Cadmium and Lead Resistance Mechanisms in Bacteria and Their Contribution to Biosensing. Ph.D. Thesis.

[68] Nies D.H. (2003). Efflux-mediated heavy metal resistance in prokaryotes. FEMS Microbiol. Rev..

[69] Borremans B., Hobman J., Provoost A., Brown N., van Der Lelie D. (2001). Cloning and functional analysis of thepbr lead resistance determinant of *Ralstonia metallidurans* CH34. J. Bacteriol..

[70] Xu L., Xu X.-W., Meng F.-X., Huo Y.-Y., Oren A., Yang J.-Y., Wang C.-S. (2013). *Halomonas zincidurans sp*. nov., a heavy-metal-tolerant bacterium isolated from the deep-sea environment. Int. J. Syst. Evol. Microbiol..

[71] Freedman Z., Zhu C., Barkay T. (2012). Mercury resistance and mercuric reductase activities and expression among chemotrophic thermophilic Aquificae. Appl. Environ. Microbiol..

[72] Silver S., Phung L.T. (2013). Bacterial Mercury resistance proteins. Encycl. Met..

[73] Zhou P., Huo Y.-Y., Xu L., Wu Y.-H., Meng F.-X., Wang C.-S., Xu X.-W. (2015). Investigation of mercury tolerance in *Chromohalobacter israelensis* DSM 6768T and *Halomonas zincidurans* B6T by comparative genomics with *Halomonas xinjiangensis* TRM 0175T. Mar. Genom..

[74] Boyd E., Barkay T. (2012). The mercury resistance operon: From an origin in a geothermal environment to an efficient detoxification machine. Front. Microbiol..

[75] Viti C., Marchi E., Decorosi F., Giovannetti L. (2014). Molecular mechanisms of Cr (VI) resistance in bacteria and fungi. FEMS Microbiol. Rev..

[76] Voica D.M., Bartha L., Banciu H.L., Oren A. (2016). Heavy metal resistance in halophilic Bacteria and Archaea. FEMS Microbiol. Lett..

[77] Branco R., Chung A.P., Johnston T., Gurel V., Morais P., Zhitkovich A. (2008). The chromate-inducible chrBACF operon from the transposable element TnOtChr confers resistance to chromium (VI) and superoxide. J. Bacteriol..

[78] Ramos J.L., Gallegos M.T., Marqués S., Ramos-González M.I., Espinosa-Urgel M., Segura A. (2001). Responses of Gram-negative bacteria to certain environmental stressors. Curr. Opin. Microbiol..

[79] Raivio T.L., Popkin D.L., Silhavy T.J. (1999). The Cpx envelope stress response is controlled by amplification and feedback inhibition. J. Bacteriol..

[80] Alba B.M., Gross C.A. (2004). Regulation of the *Escherichia coli* σ E-dependent envelope stress response. Mol. Microbiol..

[81] Cezairliyan B.O., Sauer R.T. (2007). Inhibition of regulated proteolysis by RseB. Proc. Natl. Acad. Sci. USA.

[82] Lushchak V.I. (2011). Environmentally induced oxidative stress in aquatic animals. Aquat. Toxicol..

[83] Chiang S.M., Schellhorn H.E. (2012). Regulators of oxidative stress response genes in *Escherichia coli* and their functional conservation in bacteria. Arch. Biochem. Biophys..

[84] Shiu C.-T., Lee T.-M. (2005). Ultraviolet-B-induced oxidative stress and responses of the ascorbate–glutathione cycle in a marine macroalga *Ulva Fasciata*. J. Exp. Bot..

[85] Lesser M.P. (2006). Oxidative stress in marine environments: Biochemistry and physiological ecology. Annu. Rev. Physiol..

[86] Verlecar X., Jena K., Chainy G. (2007). Biochemical markers of oxidative stress in *Perna viridis* exposed to mercury and temperature. Chem. Biol. Interact..

[87] Maleki F., Afra Khosravi A.N., Taghinejad H., Azizian M. (2016). Bacterial heat shock protein activity. Clin. Diagn Res. JCDR.

[88] Moat A.G., Foster J.W., Spector M.P. (2003). Microbial Physiology.

[89] Masip L., Veeravalli K., Georgiou G. (2006). The many faces of glutathione in bacteria. Antioxid. Redox Signal..

[90] Zhang H., Li X., Xie Y., Jin J., Liu H., Gao X., Xiong L. (2018). Carbon starvation-induced lipoprotein Slp directs the synthesis of catalase and expression of OxyR regulator to protect against hydrogen peroxide stress in *Escherichia coli*. BioRxiv.

[91] Koga T., Takumi K. (1995). Nutrient starvation induces cross protection against heat, osmotic, or H_2_O_2_ challenge in *Vibrio parahaemolyticus*. Microbiol. Immunol..

