# Supplementary material for: Life in High Salt Concentrations with Changing Environmental Conditions: Insights from Genomic and Phenotypic Analysis of Salinivibrio sp
Source: Microorganisms. 2019 Nov 19;7(11):577. doi: 10.3390/microorganisms7110577 (PMC6920786; doi:10.3390/microorganisms7110577)
Supplement: Supplementary file 1 [file microorganisms-07-00577-s001.pdf]

Supplementary table 1: Genome sequences accession numbers used in the present study

Species	Genome	Reference
S. costicola subsp. *alcaliphilus*	MUFR00000000	López-Hermoso et al., 2017
S. costicola subsp. *costicola*	AQOF00000000	López-Hermoso et al., 2017
S. costicola subsp. *vallismortis*	MUFQ00000000	López-Hermoso et al., 2017
S. proteolyticus	MUFP00000000	López-Hermoso et al., 2017
S. sharmensis	MUFC00000000	López-Hermoso et al., 2017
S. siamensis	MUFB00000000	López-Hermoso et al., 2017
S. kushneri AL184^T	MUEK00000000	López-Hermoso et al., 2017
S. kushneri IB560	MUEM00000000	López-Hermoso et al., 2017
S. kushneri IB563	MUEN00000000	López-Hermoso et al., 2017
S. kushneri IC202	MUEO00000000	López-Hermoso et al., 2017
S. kushneri IC317	MUEP00000000	López-Hermoso et al., 2017
S. kushneri MA421	MUER00000000	López-Hermoso et al., 2017
S. kushneri ML277	MUEL00000000	López-Hermoso et al., 2017
S. kushneri ML318	MUEQ00000000	López-Hermoso et al., 2017
S. kushneri ML328A	MUEU00000000	López-Hermoso et al., 2017
S. kushneri ML331	MUEV00000000	López-Hermoso et al., 2017
S. socompensis	AQOE01000016	Gorriti et al 201

Supplementary table 2: Biochemical characteristics of *Salinivibrio* sp. HTSP and its closely relative strains reported by Mellado et al [7], Romano et al [11], Amoozegar et al [85], Chamroensaksri et al [86], López-Hermoso et al [90], and Galisteo etal [8]

	HTSP	***S. kushneri***	***S. costcola* subsp. *costicola***	***S.costicola* subsp. *alcaliphilus***	***Salinivibrio socompensis***	***S. proteolyticus***	***S.sharmensis***	***S.siamensis***
Colony morphology	Circular with entire edge	Circular with entire edges	circular ,convex,opaque	round with entire edges	circular, with entire edges,convex	Round with entire edges,smooth, slighty convex	Round,bright	Circular,con v ex,opaque
Colony pigmentation	cream	cream	cream	creamy-pink	Cream-pink	Creamy white	creamy	Cream
Cell morphology	curved rods	curved rods	curved rods	curved rods	Curved rods	curved rods	curved rods	curved rods
colony size	3-3.5 μm X 0.2-0.5 μm	0.5 X2.5-3.2 μm	0.5X 1.5-3.2μm	0.5_0.6X3.2-3.5μm	3mm	0.5-0.6X 1.0-2.3μm	0.4_0.6 X3.0-3.5μm	0.4-0.5X2-8μm
Gram staining	Gram negative	Gram negative	Gram negative	Gram negative	Gram negative	Gram negative	Gram negative	Gram negative
Endospore	No	No	No	No	No	No	No	No
Motility	Yes	Yes	Yes	Yes	yes	Yes	Yes	Yes
Temperature range (°C)	18-45°C	17-49°C	5-45°C	10-40°C	10-42°C	10-45°C	25-40°C	10-45°C
pH range (otimum pH)	5-10	5-10	5-10	7-10.5	6-10	5.0-9.5	6-11	5-9
NaCl range (optimum) %, w/v	2.5-20	2_20	0.5 _10	2_25	3–20	1_1	6_16	1_17
Growth in the absence of NaCl	NO	NO	NO	NO	No	NO	NO	NO
Anaerobic fermantative growth	+	+	+	–	+	+	+	+
**Substrate and Acid production**								
Glucose	+	+	+	+	+	+	+	–
Lactose	–	+	NA	NA	+	–	–	–

Maltose	+	+	NA	NA	_	+	+	NA
Sucrose	+	_	+	+	+	_	+	+
Nitrate reduction	_	_	_	+	+	_	+	_
Indole	_	_	_	NA		_	NA	_
MR	+	_	_	+	+	_	_	_
VP	_	+	+	+		+	+	+
Citrate	_	_	NA	NA	+	_	NA	_
Starch	_	_	_	_	_	+	_	+
gelatin	NA	+	+	+	_	+	+	+
caesin	+	+	+	+	_	+	+	+
Catalase	+	+	+	+	+	+	+	+
Oxidase	+	+	+	+	+	+	+	+
H2S production	_	NA	NA	NA	_	NA	+	NA
GC % content (Genome)	50.59	50.2-50.9	49.4_50.5	49.3	49.5	49.5	51	49
Genome size (MB)	3.5	3.38	3.38	3.38	3.40	3.60	3.32	3.44

Supplementary Table 3: Genomic features associated with osmotic stress response of *Salinivibrio* sp. HTSP

**Subsystem**	**SEED database**
**Potassium metabolism**	
Potassium homeostasis	FKBP-type peptidyl-prolyl cis-trans isomerase SlyD (EC 5.2.1.8)
	putative Glutathione-regulated potassium-efflux system protein KefB
	Trk system potassium uptake protein TrkA
	Potassium uptake protein TrkH
	Putative cytoplasmic protein ,probably associated with Glutathione-regulated potassium-efflux
	Potassium voltage-gated channel subfamily KQT
	Glutathione-regulated potassium-efflux system ATP-binding protein
	FKBP-type peptidyl-prolyl cis-trans isomerase FkpA precursor (EC 5.2.1.8)
	Glutathione-regulated potassium-efflux system ancillary protein KefG
	Glutathione-regulated potassium-efflux system ATP-binding protein
	Glutathione-regulated potassium-efflux system protein KefB
	Glutathione-regulated potassium-efflux system ancillary protein KefG
Hyperosmotic potassium uptake	Trk system potassium uptake protein TrkA
	Potassium uptake protein TrkH
**Osmotic stress**	
Osmoregulation	Outer membrane protein A precursor
	Glycerol uptake facilitator protein
Ectoine biosynthesis and regulation	Diaminobutyrate-pyruvate aminotransferase (EC 2.6.1.46)
	Ectoine hydroxylase (EC 1.17.-.-)
	L-ectoine synthase (EC 4.2.1.-)
	L-2,4-diaminobutyric acid acetyltransferase (EC 2.3.1.-)
	Aspartokinase (EC 2.7.2.4) associated with ectoine biosynthesis
Synthesis of osmoregulated periplasmic glucans	Phosphoglycerol transferase I (EC 2.7.8.20)
Choline and Betaine Uptake and Betaine Biosynthesis	L-proline glycine betaine ABC transport system permease protein ProV (TC 3.A.1.12.1)

	HTH-type transcriptional regulator BetI
	L-proline glycine betaine binding ABC transporter protein ProX (TC 3.A.1.12.1)
	High-affinity choline uptake protein BetT
	Choline-sulfatase (EC 3.1.6.6)
	Choline dehydrogenase (EC 1.1.99.1)
	L-proline glycine betaine ABC transport system permease protein ProW (TC 3.A.1.12.1)
	Betaine aldehyde dehydrogenase (EC 1.2.1.8)

Supplementary Table 4: General stress-related genes observed in *Salinivibrio* sp. HTSP

Catogory	Subsystem	Role
Stress Response - no subcategory	Carbon Starvation	Carbon storage regulator
Stress Response - no subcategory	Carbon Starvation	Carbon starvation protein A
Stress Response - no subcategory	Carbon Starvation	Starvation lipoprotein Slp paralog
Stress Response - no subcategory	Carbon Starvation	Stringent starvation protein A
Stress Response - no subcategory	Carbon Starvation	Stringent starvation protein B
Oxidative stress	Cluster containing Glutathione synthetase	Glutathione synthetase (EC 6.3.2.3)
Oxidative stress	Cluster containing Glutathione synthetase	Ribosomal RNA small subunit methyltransferase E (EC 2.1.1.-)
Oxidative stress	Cluster containing Glutathione synthetase	UPF0301 protein YqgE
Oxidative stress	Cluster containing Glutathione synthetase	Putative Holliday junction resolvase YggF
Oxidative stress	CoA disulfide thiol-disulfide redox system	CoA-disulfide reductase (EC 1.8.1.14)
Cold shock	Cold shock, CspA family of proteins	Cold shock protein CspD
Cold shock	Cold shock, CspA family of proteins	Cold shock protein CspA
Cold shock	Cold shock, CspA family of proteins	Cold shock protein CspE
Oxidative stress	Glutaredoxins	Glutaredoxin
Oxidative stress	Glutaredoxins	Glutaredoxin-related protein
Oxidative stress	Glutaredoxins	Periplasmic septal ring factor with murein hydrolase activity EnvC/YibP
Oxidative stress	Glutaredoxins	Glutaredoxin 1
Oxidative stress	Glutaredoxins	Cell wall endopeptidase, family M23/M37

Oxidative stress	Glutathione: Biosynthesis and gamma-glutamyl cycle	Glutathione synthetase (EC 6.3.2.3)
Oxidative stress	Glutathione: Biosynthesis and gamma-glutamyl cycle	Glutamate--cysteine ligase (EC 6.3.2.2)
Oxidative stress	Glutathione: Non-redox reactions	Glutathione S-transferase, zeta (EC 2.5.1.18)
Oxidative stress	Glutathione: Non-redox reactions	Glutathione S-transferase (EC 2.5.1.18)
Oxidative stress	Glutathione: Non-redox reactions	Uncharacterized glutathione S-transferase-like protein
Oxidative stress	Glutathione: Non-redox reactions	Lactoylglutathione lyase (EC 4.4.1.5)
Oxidative stress	Glutathione: Non-redox reactions	FIG005121: SAM-dependent methyltransferase (EC 2.1.1.-)
Oxidative stress	Glutathione: Non-redox reactions	Glutathione S-transferase, omega (EC 2.5.1.18)
Oxidative stress	Glutathione: Non-redox reactions	Glutathione S-transferase family protein
Oxidative stress	Glutathione: Non-redox reactions	Hydroxyacylglutathione hydrolase (EC 3.1.2.6)
Oxidative stress	Glutathione: Redox cycle	Glutathione reductase (EC 1.8.1.7)
Oxidative stress	Glutathione: Redox cycle	Glutaredoxin
Oxidative stress	Glutathione: Redox cycle	Glutaredoxin 1
Detoxification	Glutathione-dependent pathway of formaldehyde detoxification	S-formylglutathione hydrolase (EC 3.1.2.12)
Detoxification	Glutathione-dependent pathway of formaldehyde detoxification	S-(hydroxymethyl)glutathione dehydrogenase (EC 1.1.1.284)
Detoxification	Glutathione-dependent pathway of formaldehyde detoxification	Transcriptional regulator, LysR family, in formaldehyde detoxification operon
Heat shock	Heat shock dnaK gene cluster extended	FIG001341: Probable Fe(2+)-trafficking protein YggX
Heat shock	Heat shock dnaK gene cluster extended	Chaperone protein DnaK
Heat shock	Heat shock dnaK gene cluster extended	Glutathione synthetase (EC 6.3.2.3)
Heat shock	Heat shock dnaK gene cluster extended	Chaperone protein DnaJ
Heat shock	Heat shock dnaK gene cluster extended	Ribosomal RNA small subunit methyltransferase E (EC 2.1.1.-)
Heat shock	Heat shock dnaK gene cluster	tmRNA-binding protein SmpB

	extended	
Heat shock	Heat shock dnaK gene cluster extended	Radical SAM family enzyme, similar to coproporphyrinogen III oxidase, oxygen-independent, clustered with nucleoside-triphosphatase RdgB
Heat shock	Heat shock dnaK gene cluster extended	Heat shock protein GrpE
Heat shock	Heat shock dnaK gene cluster extended	RNA polymerase sigma factor RpoH
Heat shock	Heat shock dnaK gene cluster extended	Translation elongation factor LepA
Heat shock	Heat shock dnaK gene cluster extended	Ribosome-associated heat shock protein implicated in the recycling of the 50S subunit (S4 paralog)
Heat shock	Heat shock dnaK gene cluster extended	Nucleoside 5-triphosphatase RdgB (dHAPTP, dITP, XTP-specific) (EC 3.6.1.15)
Heat shock	Heat shock dnaK gene cluster extended	Ribosomal protein L11 methyltransferase (EC 2.1.1.-)
Heat shock	Heat shock dnaK gene cluster extended	Ribonuclease PH (EC 2.7.7.56)
Heat shock	Heat shock dnaK gene cluster extended	rRNA small subunit methyltransferase I
Oxidative stress	Oxidative stress	Iron-binding ferritin-like antioxidant protein
Oxidative stress	Oxidative stress	Redox-sensitive transcriptional activator SoxR
Oxidative stress	Oxidative stress	NnrS protein involved in response to NO
Oxidative stress	Oxidative stress	Alkyl hydroperoxide reductase subunit C-like protein
Oxidative stress	Oxidative stress	Manganese superoxide dismutase (EC 1.15.1.1)
Oxidative stress	Oxidative stress	Paraquat-inducible protein B
Oxidative stress	Oxidative stress	Superoxide dismutase [Fe] (EC 1.15.1.1)
Oxidative stress	Oxidative stress	Hydrogen peroxide-inducible genes activator
Oxidative stress	Oxidative stress	Catalase (EC 1.11.1.6)
Oxidative stress	Oxidative stress	Fumarate and nitrate reduction regulatory protein
Oxidative stress	Oxidative stress	Organic hydroperoxide resistance protein
Oxidative stress	Oxidative stress	Organic hydroperoxide resistance transcriptional regulator

Oxidative stress	Oxidative stress	Zinc uptake regulation protein ZUR
Oxidative stress	Oxidative stress	Non-specific DNA-binding protein Dps
Oxidative stress	Oxidative stress	Ferroxidase (EC 1.16.3.1)
Oxidative stress	Oxidative stress	Ferric uptake regulation protein FUR
Oxidative stress	Oxidative stress	Peroxidase (EC 1.11.1.7)
Oxidative stress	Oxidative stress	Paraquat-inducible protein A
Periplasmic Stress	Periplasmic Stress Response	Survival protein SurA precursor (Peptidyl-prolyl cis-trans isomerase SurA) (EC 5.2.1.8)
Periplasmic Stress	Periplasmic Stress Response	Sigma factor RpoE negative regulatory protein RseA
Periplasmic Stress	Periplasmic Stress Response	Outer membrane stress sensor protease DegS
Periplasmic Stress	Periplasmic Stress Response	Outer membrane protein H precursor
Periplasmic Stress	Periplasmic Stress Response	Sigma factor RpoE negative regulatory protein RseB precursor
Periplasmic Stress	Periplasmic Stress Response	Outer membrane stress sensor protease DegQ, serine protease
Oxidative stress	Protection from Reactive Oxygen Species	Peroxidase (EC 1.11.1.7)
Oxidative stress	Protection from Reactive Oxygen Species	Manganese superoxide dismutase (EC 1.15.1.1)
Oxidative stress	Protection from Reactive Oxygen Species	Superoxide dismutase [Fe] (EC 1.15.1.1)
Oxidative stress	Protection from Reactive Oxygen Species	Catalase (EC 1.11.1.6)
Oxidative stress	Redox-dependent regulation of nucleus processes	Nicotinate phosphoribosyltransferase (EC 2.4.2.11)
Oxidative stress	Redox-dependent regulation of nucleus processes	NADPH-dependent glyceraldehyde-3-phosphate dehydrogenase (EC 1.2.1.13)
Oxidative stress	Redox-dependent regulation of nucleus processes	Nicotinamidase (EC 3.5.1.19)
Oxidative stress	Redox-dependent regulation of nucleus processes	NAD-dependent protein deacetylase of SIR2 family
Oxidative stress	Redox-dependent regulation of nucleus processes	NAD-dependent glyceraldehyde-3-phosphate dehydrogenase (EC 1.2.1.12)
Stress Response - no	Hfl operon	Putative inner membrane protein YjeT (clustered with HflC)

subcategory		
Stress Response - no subcategory	Hfl operon	GTP-binding protein HflX
Stress Response - no subcategory	Hfl operon	HflC protein
Stress Response - no subcategory	Hfl operon	RNA-binding protein Hfq
Stress Response - no subcategory	Hfl operon	HflK protein
Stress Response - no subcategory	Sugar-phosphate stress regulation	SgrR, sugar-phosphate stress, transcriptional activator of SgrS small RNA
Stress Response - no subcategory	Universal stress protein family	Universal stress protein E
Stress Response - no subcategory	Universal stress protein family	Universal stress protein B
Stress Response - no subcategory	Universal stress protein family	Universal stress protein family 3
Stress Response - no subcategory	Universal stress protein family	Universal stress protein A
Stress Response - no subcategory	Phage shock protein (psp) operon	Psp operon transcriptional activator
Stress Response - no subcategory	Phage shock protein (psp) operon	Phage shock protein B
Stress Response - no subcategory	Phage shock protein (psp) operon	Phage shock protein C
Stress Response - no subcategory	Phage shock protein (psp) operon	Phage shock protein A

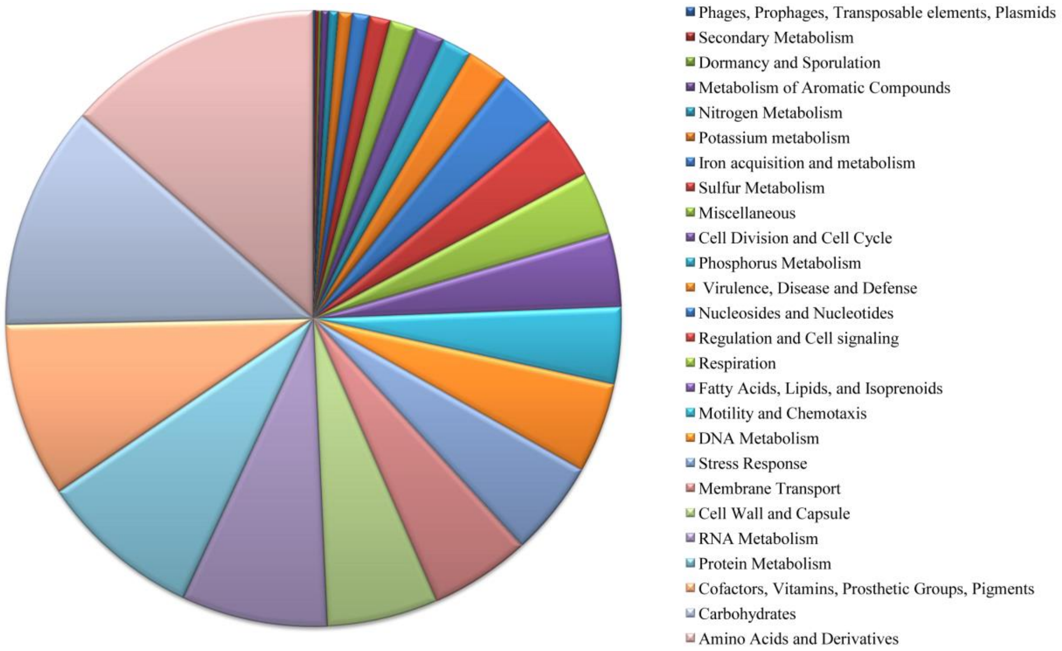

Supplementary figure 1: Subsystem category distribution statistics for *Salinivibrio* sp. HTSP

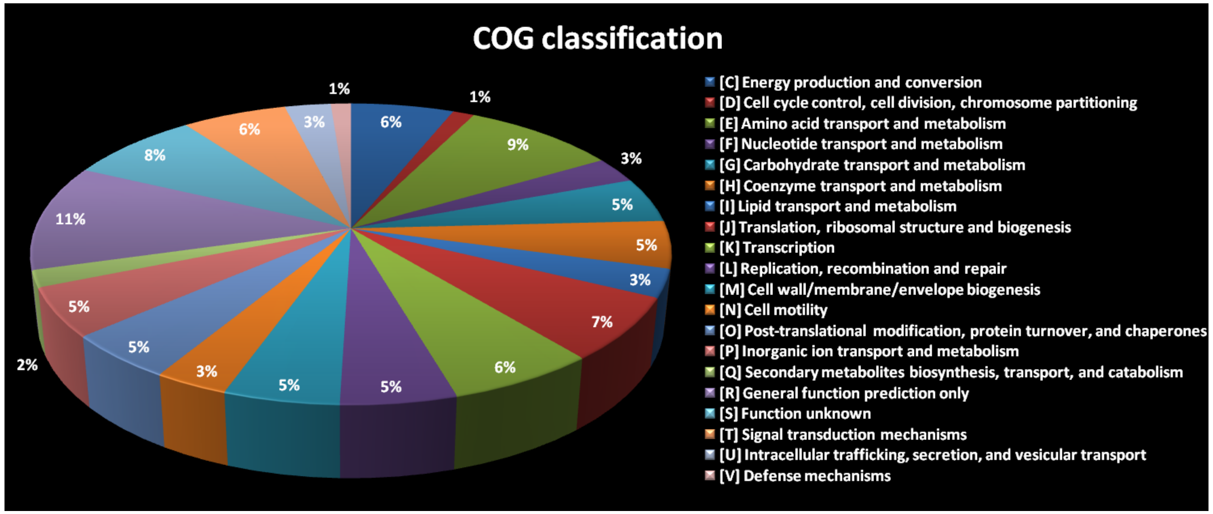

Supplementary Figure 2: COG functional classification of core orthologous gene of *Salinivibrio* sp. *HTSP*
